# Long-term Survival and Cost-effectiveness Associated With Axicabtagene Ciloleucel vs Chemotherapy for Treatment of B-Cell Lymphoma

**DOI:** 10.1001/jamanetworkopen.2019.0035

**Published:** 2019-02-22

**Authors:** Melanie D. Whittington, R. Brett McQueen, Daniel A. Ollendorf, Varun M. Kumar, Richard H. Chapman, Jeffrey A. Tice, Steven D. Pearson, Jonathan D. Campbell

**Affiliations:** 1 Department of Clinical Pharmacy, Skaggs School of Pharmacy and Pharmaceutical Sciences, University of Colorado Anschutz Medical Campus, Aurora; 2 Institute for Clinical and Economic Review, Boston, Massachusetts; 3 Department of Medicine, University of California, San Francisco

## Abstract

**Importance:**

Axicabtagene ciloleucel, a chimeric antigen receptor T-cell therapy, represents a new and potentially curative treatment option for B-cell lymphoma. It is expected to have long-term survival benefits; however, long-term survival data are limited.

**Objective:**

To estimate the long-term survival and cost-effectiveness of axicabtagene ciloleucel for treatment of relapsed or refractory B-cell lymphoma.

**Design, Setting, and Participants:**

Economic evaluation study using a survival analysis that digitized and extrapolated survival curves published in the ZUMA-1 trial (Safety and Efficacy of KTE-C19 in Adults With Refractory Aggressive Non-Hodgkin Lymphoma), which enrolled patients between November 2015 and September 2016 and had a maximum follow-up of 24 months. Five different survival models (standard parametric, flexible parametric, 2 mixture cure models, and a flexible parametric mixture model) were used to extrapolate the survival curves to a lifetime horizon from January through June 2018. A cost-effectiveness analysis, from both a trial-based and lifetime horizon, was also conducted to inform the value of this novel therapy. The model was based on data from 111 patients with B-cell lymphoma who were enrolled in the ZUMA-1 trial.

**Interventions:**

One-time administration of axicabtagene ciloleucel compared with chemotherapy.

**Main Outcomes and Measures:**

Undiscounted and discounted life-years (LYs) and quality-adjusted life-years (QALYs), total costs, and incremental costs per LY and QALY gained.

**Results:**

The modeled cohort of 111 patients started at 58 years of age. At the end of the trial, treatment with axicabtagene ciloleucel resulted in 0.48 more LYs and 0.34 more QALYs than chemotherapy, producing a cost-effectiveness estimate of $896 600 per QALY for public payers and $1 615 000 per QALY for commercial payers. Extrapolated long-term survival for patients treated with axicabtagene ciloleucel ranged from 2.83 to 9.19 discounted LYs and from 2.07 to 7.62 discounted QALYs. Incrementally, treatment with axicabtagene ciloleucel was associated with 1.89 to 5.82 discounted LYs and 1.52 to 4.90 discounted QALYs vs chemotherapy. With the use of these incremental estimates of survival, cost-effectiveness estimates ranged from $82 400 to $230 900 per QALY gained for public payers and from $100 400 to $289 000 per QALY gained for commercial payers.

**Conclusions and Relevance:**

Treatment with axicabtagene ciloleucel appears to be associated with incremental gains in survival over chemotherapy. The range in projected long-term survival was wide and reflected uncertainty owing to limited follow-up data. Cost-effectiveness is associated with long-term survival, with further evidence needed to reduce uncertainty.

## Key Points

**Question:**

What are the incremental survival gains and cost-effectiveness of axicabtagene ciloleucel vs chemotherapy for treatment of B-cell lymphoma in adults?

**Findings:**

In this economic evaluation study, a survival and cost-effectiveness analysis of ZUMA-1 trial data found that at the end of the trial (24 months), treatment with axicabtagene ciloleucel resulted in more quality-adjusted life-years than chemotherapy, producing a cost-effectiveness estimate between $896 600 and $1 615 000 per quality-adjusted life-year gained. Lifetime survival gains for treatment with axicabtagene ciloleucel ranged from 1.52 to 4.90 more quality-adjusted life-years, and a cost-effectiveness estimate ranged from $82 400 to $289 000 per quality-adjusted life-year gained over a lifetime horizon.

**Meaning:**

Treatment with axicabtagene ciloleucel appears to be associated with positive, yet uncertain, gains in survival compared with chemotherapy, and its cost-effectiveness is associated with long-term survival.

## Teaser

This survival and cost-effectiveness analysis study uses survival curves from the ZUMA-1 trial (Safety and Efficacy of KTE-C19 in Adults With Refractory Aggressive Non-Hodgkin Lymphoma) to estimate long-term survival and cost-effectiveness associated with axicabtagene ciloleucel, a chimeric antigen receptor T-cell therapy, vs chemotherapy for treatment of relapsed or refractory B-cell lymphoma in adults.

## Introduction

Diffuse, large B-cell lymphoma is the most common subtype of non-Hodgkin lymphoma in the United States, accounting for 30% to 40% of all non-Hodgkin lymphoma cases.^1,2^ Owing to the aggressive attack on lymph nodes outside of the lymphatic system in patients with diffuse, large B-cell lymphoma, the outlook for those patients whose condition fails to respond to initial chemotherapy cycles is poor. Even if a patient’s condition responds to second-line chemotherapy and the patient completes autologous stem cell transplantation, 5-year progression-free survival is estimated to be only 10% to 20%.^3,4,5^ The recent development and approval of axicabtagene ciloleucel, a chimeric antigen receptor T-cell (CAR-T) therapy, represents a new and potentially curative option.^6^ The overall response rate for treatment with axicabtagene ciloleucel in its pivotal trial was 82%, with estimates of overall survival at 6 months of 80%.^7^

The trial evidence on survival is promising, but follow-up has been limited in part because of the US Food and Drug Administration’s breakthrough therapy designation and consequent accelerated approval timeline.^6^ The median follow-up is 15.4 months, and the maximum follow-up is 24 months, although benefits may persist for a lifetime.^7^ The purpose of the present study was to estimate long-term survival gains for patients treated with axicabtagene ciloleucel using trial evidence and recommended long-term survival extrapolation techniques.^8,9,10,11^ Trial-based and projected long-term survival estimates were then used to calculate the cost-effectiveness of treatment with axicabtagene ciloleucel to inform the value of this novel therapy.

## Methods

This analysis was conducted according to the Consolidated Health Economic Evaluation Reporting Standards (CHEERS) guideline. The study design and analysis are described succinctly herein, but for the purposes of full model replication, specific model details that are less important for understanding the results and their interpretation are available in the eAppendix in the Supplement. The Colorado Multiple Institutional Review Board in Aurora determined that this study was not considered human subjects research because it used already available data, did not include patient-level data, and did not interact with patients, and thus study approval and informed patient consent were not needed.

### Study Design

The Kaplan-Meier curves published in the axicabtagene ciloleucel pivotal trial ZUMA-1 (Safety and Efficacy of KTE-C19 in Adults With Refractory Aggressive Non-Hodgkin Lymphoma),^7^ which enrolled 111 patients between November 2015 and September 2016 and had a median follow-up of 15.4 months, were first digitized using the algorithm by Guyot and colleagues^12^ to impute patient-level time-to-event data. We extracted data points from the digitized copies of the published survival curves^13^ and then used the extracted values, the number of surviving patients at each time interval, and maximum likelihood functions to estimate the underlying individual patient data. Survival at the end of the trial time horizon, which was 24 months, was calculated for both treatment with axicabtagene ciloleucel and chemotherapy to inform the incremental survival gains associated with axicabtagene ciloleucel at the end of the trial. At the end of the trial, the survival curves for treatment with axicabtagene ciloleucel and chemotherapy had not yet converged, suggesting continued benefit of treatment with axicabtagene ciloleucel over chemotherapy even after the trial time horizon. We extrapolated the trial survival curves over a lifetime horizon to fully capture the differences between treatment with axicabtagene ciloleucel and chemotherapy and to estimate the lifetime incremental benefit associated with axicabtagene ciloleucel. To generate a range in possible long-term survival scenarios, 5 survival models were fit to the published survival curves.^7,14^ Variation in long-term survival assumptions was captured by these 5 different models to produce a range of long-term survival estimates. For each survival model, we estimated the mean discounted and undiscounted life-years (LYs) and quality-adjusted life-years (QALYs). Long-term survival for patients receiving comparator chemotherapies was also estimated to calculate incremental survival gains associated with axicabtagene ciloleucel.

Total costs and cost-effectiveness estimates were then calculated to inform the value of axicabtagene ciloleucel using a cost-effectiveness analysis. The cost-effectiveness analysis model structure included a short-term decision tree and a long-term semi-Markov partitioned survival model (eFigure in the Supplement). The decision tree calculated the costs and consequences from treatment initiation to assessment of response, which was approximately 1 month.^15^ From the decision tree, patients moved to the semi-Markov partitioned survival model, where they were then tracked for a trial-based time horizon of 24 months and a lifetime horizon. Cost-effectiveness estimates were presented over a short-term time horizon (ie, trial-based time horizon of 24 months) and over a lifetime horizon. Survival extrapolation and the cost-effectiveness analyses were conducted from January through June 2018.

### Statistical Analysis

#### Long-term Survival Modeling

We fit 5 different survival models to the published progression-free and overall survival curves^7,14^: (1) standard parametric model, (2) flexible parametric model, (3) mixture cure model assuming those alive and responding to treatment at the end of the trial were cured, (4) mixture cure model assuming all of those alive at the end of the trial (regardless of response status) were cured, and (5) flexible parametric mixture model. The survival models were then used to extrapolate survival beyond the observed data reported in the trials. Features of each survival model, including differences between each modeling method, are described in Table 1. Survival curve digitization was accomplished using R, version 3.5.2 (The R Foundation).

**Table 1.** Characteristics of Each Survival Model

Feature of Survival Extrapolation	Standard Parametric^a^	Flexible Parametric^b^	Mixture Cure 1^c^	Mixture Cure 2^d^	Flexible Parametric Mixture^e^
Parametric curve for downward slope of survival curve	✓	✓	✓	✓	✓
Knot introduced in parametric curve at survival curve flattening		✓	✓	✓	✓
Alive and not responding at end of follow-up, died within 2 mo	✓	✓	✓		✓
Separate models for cured vs not cured			✓	✓	
Excess death modeled for long-term survivors					✓

^a^ Fit a parametric function to the published curve and extrapolated the parametric function to a lifetime horizon.

^b^ Introduced a knot in the parametric function at the point where the published survival curve flattened.

^c^ Introduced a knot in the parametric function at the point where the published survival curve ended; assumed everyone alive and responding to treatment at end of trial follow-up was cured.

^d^ Introduced a knot in the parametric function at the point where the published survival curve ended; assumed everyone alive at end of trial follow-up was cured.

^e^ Introduced a knot in the parametric function at the point where the published survival curve flattened and modeled excess death among long-term survivors.

#### Cost-effectiveness Model Structure

A decision analytic model was used to aggregate all costs and outcomes expected from treatment with axicabtagene ciloleucel and from chemotherapy. Patient survival, quality-adjusted survival, and health care costs were estimated over both a trial-based time horizon and a lifetime horizon for treatment with axicabtagene ciloleucel and for chemotherapy. Key model assumptions are provided in eTable 1 in the Supplement. The model tracked patients from treatment initiation (considered leukapheresis for axicabtagene ciloleucel) until death. The modeled cohort started at an age of 58 years.^15^ The estimates from the survival modeling were used as inputs in the decision analytic model. Other model inputs can be found in eTables 2 to 12 in the Supplement.

Cost-effectiveness estimates were calculated from 2 different payer perspectives: a public payer perspective and a commercial payer perspective. The difference between perspectives was primarily associated with the assumed hospital markup for treatment acquisition. For the public payer perspective, no hospital markup was assumed for treatments administered on an inpatient basis, but a markup of treatment acquisition cost plus 6% was assumed for treatments administered on an outpatient basis.^16^ For the commercial payer perspective, a hospital markup of treatment acquisition price plus 152%^17^ was assumed for treatments administered on an inpatient basis, and a markup of treatment acquisition price plus 28%^17^ was assumed for treatments administered on an outpatient basis. Based on comments from CAR-T contracting experts, markups for treatment with axicabtagene ciloleucel were capped at $100 000 to account for the notion that some facilities may not negotiate a markup (ie, they will manage CAR-T as a pass-through) while other facilities may charge a markup. Analyses of both the survival models and the cost-effectiveness models were conducted using Microsoft Excel, 2016 (Microsoft Corp).

## Results

### Short-term Survival and Cost-effectiveness

The modeled cohort of 111 patients started at 58 years of age. Incremental survival was first calculated at the end of the trial time horizon, which was approximately 24 months. A patient who had received axicabtagene ciloleucel gained a mean of 1.39 LYs and 0.91 QALYs at the end of the trial time horizon. This can be contrasted with a mean of 0.91 LYs and 0.57 QALYs gained at the end of the trial time horizon for a patient who had been treated with chemotherapy. Therefore, treatment with axicabtagene ciloleucel was associated with an incremental survival gain of 0.48 LYs and 0.34 QALYs at the 24-month trial time horizon. Although associated with survival gains, treatment with axicabtagene ciloleucel also had higher costs at the end of the trial time horizon. The mean total cost for a patient treated with axicabtagene ciloleucel was approximately $521 000. The mean cost for a patient treated with chemotherapy was $403 000 less. This resulted in a cost-effectiveness estimate for treatment with axicabtagene ciloleucel at the end of the trial time horizon of $1 615 000 per QALY gained from the commercial payer perspective and $896 600 per QALY gained from the public payer perspective.

### Long-term Survival and Cost-effectiveness

The long-term survival outcomes from the 5 different survival models are provided in Table 2 and Table 3. The incremental improvements for treatment with axicabtagene ciloleucel compared with chemotherapy ranged from 1.52 more discounted QALYs (1.89 more discounted LYs) in the standard parametric model to 4.90 more discounted QALYs (5.82 more discounted LYs) for the mixture cure model that assumed all those patients alive at the end of follow-up were long-term survivors. Across the 5 survival models, long-term survival for patients who received axicabtagene ciloleucel ranged from 2.83 to 9.19 discounted LYs (3.58-13.45 undiscounted LYs) and from 2.07 to 7.62 discounted QALYs (2.69-11.12 undiscounted QALYs). By contrast, long-term survival for patients who received chemotherapy ranged from 0.94 to 3.37 discounted LYs (0.96-4.73 undiscounted LYs) and from 0.55 to 2.72 discounted QALYs (0.57-3.83 undiscounted QALYs).

**Table 2.** Discounted Costs, QALYs, and Cost-effectiveness for Each Survival Model, Public Payer Perspective

Model Outcome and Treatment	Standard Parametric^a^	Flexible Parametric^b^	Mixture Cure 1^c^	Mixture Cure 2^d^	Flexible Parametric Mixture^e^
Total costs, $					
Axicabtagene ciloleucel	459 700	519 400	529 900	554 700	474 500
Chemotherapy	108 600	143 500	147 600	151 200	126 400
Incremental	351 100	375 900	382 300	403 500	348 100
Discounted total LYs					
Axicabtagene ciloleucel	2.83	7.35	7.66	9.19	5.30
Chemotherapy	0.94	3.21	3.17	3.37	2.40
Incremental	1.89	4.14	4.49	5.82	2.90
Discounted total QALYs					
Axicabtagene ciloleucel	2.07	5.84	6.34	7.62	4.14
Chemotherapy	0.55	2.46	2.55	2.72	1.80
Incremental	1.52	3.38	3.79	4.90	2.34
Cost-effectiveness					
$/LY	185 800	90 800	85 100	69 300	120 000
$/QALY	230 900	111 200	100 900	82 400	148 800

Abbreviations: LY, life-year; QALY, quality-adjusted life-year.

^a^ Fit a parametric function to the published curve and extrapolated the parametric function to a lifetime horizon.

^b^ Introduced a knot in the parametric function at the point where the published survival curve flattened.

^c^ Introduced a knot in the parametric function at the point where the published survival curve ended; assumed everyone alive and responding to treatment at end of trial follow-up was cured.

^d^ Introduced a knot in the parametric function at the point where the published survival curve ended; assumed everyone alive at end of trial follow-up was cured.

^e^ Introduced a knot in the parametric function at the point where the published survival curve flattened and modeled excess death among long-term survivors.

**Table 3.** Discounted Costs, QALYs, and Cost-effectiveness for Each Survival Model, Commercial Payer Perspective

Model Outcome and Treatment	Standard Parametric^a^	Flexible Parametric^b^	Mixture Cure 1^c^	Mixture Cure 2^d^	Flexible Parametric Mixture^e^
Total costs, $					
Axicabtagene ciloleucel	554 000	613 600	624 200	648 900	568 800
Chemotherapy	114 500	149 400	152 500	157 000	132 300
Incremental	439 500	464 200	471 700	491 900	436 500
Discounted total LYs					
Axicabtagene ciloleucel	2.83	7.35	7.66	9.19	5.30
Chemotherapy	0.94	3.21	3.17	3.37	2.40
Incremental	1.89	4.14	4.49	5.82	2.90
Discounted total QALYs					
Axicabtagene ciloleucel	2.07	5.84	6.34	7.62	4.14
Chemotherapy	0.55	2.46	2.55	2.72	1.80
Incremental	1.52	3.38	3.79	4.90	2.34
Cost-effectiveness					
$/LY	233 500	112 100	105 000	84 500	150 500
$/QALY	289 000	137 300	124 200	100 400	186 500

Abbreviations: LY, life-year; QALY, quality-adjusted life-year.

^a^ Fit a parametric function to the published curve and extrapolated the parametric function to a lifetime horizon.

^b^ Introduced a knot in the parametric function at the point where the published survival curve flattened.

^c^ Introduced a knot in the parametric function at the point where the published survival curve ended; assumed everyone alive and responding to treatment at end of trial follow-up was cured.

^d^ Introduced a knot in the parametric function at the point where the published survival curve ended; assumed everyone alive at end of trial follow-up was cured.

^e^ Introduced a knot in the parametric function at the point where the published survival curve flattened and modeled excess death among long-term survivors.

Table 2 and Table 3 also present the total costs and cost-effectiveness estimates comparing treatment with axicabtagene ciloleucel with chemotherapy for each survival model. Table 2 presents the estimates from a public payer perspective, and Table 3 presents the estimates from a commercial payer perspective. Using these estimates of survival, we found that the cost-effectiveness estimates ranged from $82 400 to $230 900 per QALY gained for public payers and from $100 400 to $289 000 per QALY gained for commercial payers. The cost-effectiveness estimates from the commercial payer perspective were higher than the respective cost-effectiveness estimates from the public payer perspective because of the higher assumed hospital markup for commercial payers.

## Discussion

We first calculated survival and cost-effectiveness estimates at the end of the ZUMA-1 trial follow-up. This analysis did not require extrapolation and thus did not require assumptions regarding long-term survival. However, this analysis likely drastically underestimated the survival and value of treatment with axicabtagene ciloleucel because benefits were expected to persist beyond the trial follow-up. The trial results suggested continued benefit beyond the trial follow-up, as evidenced by separation in the survival curves between treatment with axicabtagene ciloleucel and chemotherapy even at the end of the trial. Thus, we do not believe the survival and cost-effectiveness estimates from the trial-based time horizon adequately represented the full value of treatment with axicabtagene ciloleucel. However, the results of the present short-term analysis do highlight how long-term survival and long-term cost-effectiveness are dependent on survival extrapolation beyond the trial time horizon.

Among the long-term survival model scenarios, the standard parametric survival model resulted in the fewest mean incremental QALYs gained for treatment with axicabtagene ciloleucel compared with chemotherapy. This was expected because the standard parametric modeling approach does not account for flattening in survival curves. When the survival curves exhibit flattening (ie, the survival curves level off), as was observed in the published axicabtagene ciloleucel treatment curves,^7^ standard parametric survival modeling likely underestimates long-term survival. Therefore, incremental findings from the standard parametric survival model should be interpreted as a possible lower bound of long-term survival given current survival assumptions. Novel survival models that are able to account for survival curve flattening, including the flexible parametric, mixture cure, and flexible parametric mixture cure models, are likely more appropriate with potentially curative therapies. The novel survival models generated a range from 2.34 to 4.90 incremental QALYs for treatment with axicabtagene ciloleucel compared with chemotherapy.

Because the standard parametric model generated the smallest incremental difference in QALYs gained between treatment with axicabtagene ciloleucel and chemotherapy, this model resulted in the least favorable cost-effectiveness estimate. Conversely, the mixture cure models generated the most favorable cost-effectiveness estimates. This result was driven by the largest incremental difference in QALY gains for treatment with axicabtagene ciloleucel compared with chemotherapy generated by the mixture cure models.

Using the extrapolated estimates of lifetime survival from 5 survival extrapolation approaches, we found that incremental cost-effectiveness estimates ranged from $82 400 to $230 900 per QALY gained for public payers and from $100 400 to $289 000 per QALY gained for commercial payers. This wide range was produced from different assumptions around long-term survival. Without long-term evidence, selection of which survival model is the most accurate remains unknown; however, the present research provides a range for the estimated long-term survival based on the current evidence available.

A recent study by Roth and colleagues^18^ used a mixture cure model to extrapolate survival observed from phase 1 axicabtagene ciloleucel evidence and from the SCHOLAR-1 (international, multicohort, retrospective non-Hodgkin lymphoma research) study. Their survival extrapolation approach most closely aligns with our mixture cure model 2. Their model estimates lifetime costs of $553 000 for treatment with axicabtagene ciloleucel and $173 000 for chemotherapy. Our mixture cure model 2 estimated similar lifetime costs for both the axicabtagene ciloleucel arm and the chemotherapy arm. Their model estimates 7.7 QALYs for treatment with axicabtagene ciloleucel and 1.1 QALYs for chemotherapy.^18^ Our mixture cure model estimated similar QALYs for treatment with axicabtagene ciloleucel but more QALYs for chemotherapy. The large differences in the 2 models are in the chemotherapy arms, not the axicabtagene ciloleucel arms as implied by Roth and colleagues.^18^ Therefore, our mixture cure model generated a slightly higher cost-effectiveness estimate ($82 400 per QALY) than what Roth et al^18^ reported ($58 000 per QALY). Because of the uncertainty in long-term survival and the reliance on long-term survival extrapolation and corresponding assumptions, we argue that it is important to generate and present the results from multiple potential survival models that have differing but plausible assumptions. The quantification of the results from different plausible models is synonymous with the concept of structural uncertainty, an uncertainty domain often missing from cost-effectiveness analyses.^19^ A structural uncertainty approach allows for a range of potential long-term survival and cost-effectiveness estimates and may result in a more accurate presentation of the overall uncertainty.

### Limitations

The present analysis was limited primarily by the lack of comparative evidence available for treatment with axicabtagene ciloleucel because survival evidence was only available from a single-arm trial. Furthermore, long-term follow-up on progression-free and overall survival was limited. Although we lacked access to patient-level evidence, mixture model 2 projected similar lifetime QALYs for treatment with axicabtagene ciloleucel compared with a patient-level projected analysis.^18^ With or without patient-level data, assumptions remain associated with the extrapolation of the trial survival curve and the time point at which long-term survivors would be considered effectively cured. Uncertainty in these assumptions was tested using 5 different survival extrapolation scenarios. Also, mechanisms for the payment of the treatment with axicabtagene ciloleucel are still largely unknown (eg, bundled payment vs fee-for-service or the amount of hospital markup), requiring assumptions regarding the costs and payment of these therapies. These uncertainties were partially addressed by presenting cost-effectiveness findings separately for the public payer and commercial payer perspectives.

## Conclusions

Given the evidence available at this time, treatment with axicabtagene ciloleucel appears to be associated with positive but uncertain gains in survival compared with chemotherapy over a lifetime horizon. Under certain long-term survival assumptions, treatment with axicabtagene ciloleucel also appears to be cost-effective. Data from patients receiving this novel CAR-T therapy should continue to be collected to reduce the uncertainty in long-term survival and thus cost-effectiveness estimates.

## Notes

Supplement. **eAppendix.** Model Structure **eFigure.** Model Schematic **eTable 1.** Key Model Assumptions **eTable 2.** Treatment Regimens **eTable 3.** Response to Treatment **eTable 4.** Source of Kaplan-Meier Curves to Calculate Transition Probabilities **eTable 5.** Receipt of Stem Cell Transplantation **eTable 6.** Included Adverse Event Rates **eTable 7.** Model Health State Utilities **eTable 8**. Treatment-Related Disutilities **eTable 9.** Treatment Acquisition Costs for B-Cell Lymphoma Cohort **eTable 10.** Unit Costs for Health Care Utilization **eTable 11.** Administration and Monitoring **eTable 12.** Adverse Event Unit Costs **eReferences**
