# Supplementary material for: Long-term Survival and Cost-effectiveness Associated With Axicabtagene Ciloleucel vs Chemotherapy for Treatment of B-Cell Lymphoma
Source: JAMA Netw Open. 2019 Feb 22;2(2):e190035. doi: 10.1001/jamanetworkopen.2019.0035 (PMC6484589; doi:10.1001/jamanetworkopen.2019.0035)
Supplement: Supplement. — eAppendix. Model Structure eFigure. Model Schematic eTable 1. Key Model Assumptions eTable 2. Treatment Regimens eTable 3. Response to Treatment eTable 4. Source of Kaplan-Meier Curves to Calculate Transition Probabilities eTable 5. Receipt of Stem Cell Transplantation eTable 6. Included Adverse Event Rates eTable 7. Model Health State Utilities eTable 8. Treatment-Related Disutilities eTable 9. Treatment Acquisition Costs for B-Cell Lymphoma Cohort eTable 10. Unit Costs for Health Care Utilization eTable 11. Administration and Monitoring eTable 12. Adverse Event Unit Costs eReferences [file jamanetwopen-2-e190035-s001.pdf]

Supplementary Online Content

Whittington MD, McQueen RB, Ollendorf DA, et al. Long-term survival and cost-effectiveness associated with axicabtagene ciloleucel vs chemotherapy for treatment of B-cell lymphoma. *JAMA Netw Open*. 2019;2(2):e190035. doi:10.1001/jamanetworkopen.2019.0035

eAppendix. Model Structure

eFigure. Model Schematic

eTable 1. Key Model Assumptions

eTable 2. Treatment Regimens

eTable 3. Response to Treatment

eTable 4. Source of Kaplan-Meier Curves to Calculate Transition Probabilities

eTable 5. Receipt of Stem Cell Transplantation

eTable 6. Included Adverse Event Rates

eTable 7. Model Health State Utilities

eTable 8. Treatment-Related Disutilities

eTable 9. Treatment Acquisition Costs for B-Cell Lymphoma Cohort

eTable 10. Unit Costs for Health Care Utilization

eTable 11. Administration and Monitoring

eTable 12. Adverse Event Unit Costs

eReferences

This supplementary material has been provided by the authors to give readers additional information about their work.

© 2019 Whittington MD et al. *JAMA Network Open*.

eAppendix. Model Structure

Methodology was adapted from the Institute for Clinical and Economic Review (ICER) final report on CAR-T therapies.[1] The decision analytic model structure included a short-term decision tree and a long-term semi-Markov partitioned-survival model. The decision tree calculated the costs and consequences from treatment initiation to assessment of response, which was approximately one month.[2] From the decision tree, patients moved to the semi-Markov partitioned-survival model where they were then tracked for a trial-based time horizon of 24 months and a lifetime time horizon. The purpose of the decision tree was to stratify the cohort by which treatment they ended up receiving, because the model starts at treatment initiation (considered leukapheresis for axicabtagene ciloleucel). Further, the decision tree allowed for allocation of upfront costs by treatment and the stratification of the cohort by response status.

For the decision tree, the CAR-T arm included patients who were eligible for axicabtagene ciloleucel and underwent leukapheresis. At the first decision tree event node of the CAR-T arm, patients had three possibilities: 1) continue with axicabtagene ciloleucel after undergoing leukapheresis to receive the infusion; 2) discontinue axicabtagene ciloleucel (before infusion but after leukapheresis) because of adverse events or manufacturing failures; or 3) die before receiving the infusion. Those who discontinued axicabtagene ciloleucel due to adverse events were assumed to not be able to tolerate other active therapies and therefore transitioned to receive no further antilymphomic therapy (i.e., palliative care only). Those who discontinued axicabtagene ciloleucel due to manufacturing failures were assumed to receive chemotherapy. Responses were assessed for patients who received the axicabtagene ciloleucel infusion (second event node of decision tree), which could be: alive and responding to treatment; alive and not responding to treatment; or dead before assessment of response. The model was flexible enough to allow for patients to receive or not receive stem cell transplantation (third event node of decision tree) based on percentages reported in available evidence. The decision tree's comparator arm followed a similar pathway to the CAR-T arm, tracking the patient from chemotherapy treatment initiation through assessment of response and receipt of stem cell transplantation.

From the decision tree, the cohort was assigned to three mutually exclusive health states in a semi-Markov partitioned survival model that followed patients for the remainder of their lifetime using survival curve evidence. The three health states included: 1) alive and responding to treatment, 2) alive and not responding to treatment, and 3) death from DLBCL or other causes.

Patients transitioned between states during predetermined cycles (one month) over a lifetime time horizon. The "alive and responding to treatment" health state included all patients who were alive and responding to treatment (complete or partial responders). The "alive and not responding to treatment" health state included all patients who were alive that did not respond to treatment or relapsed after previously responding to treatment. Patients in the "alive and not responding to treatment" health state remained in this health state until they died from their modeled B-cell malignancy or other causes. Patients not responding to treatment received palliative chemotherapy. End-of-life hospice care costs were assigned to each death event. Health state occupancy was derived using five different partitioned survival techniques involving

© 2019 Whittington MD et al. *JAMA Network Open*.

the direct extrapolation of progression-free survival (PFS) and overall survival (OS) Kaplan-Meier curves:

alive and responding to treatment (t)=P(PFS, t)

alive and not responding to treatment (t)=(P(OS, t)-P(PFS, t)

death (t) = 1-P(OS, t)

Although the decision tree separated the cohort based on response status, survival curves were not available stratified by response status for all treatments. Further, definitions of response may vary between treatments; thus, survival curves were based on aggregated cohort data and not stratified by response status. Thus, in our models, there is no structural link between response status and survival. The models were developed in Microsoft Excel.

The analysis provided results from both the public and commercial payer perspective and focused on direct medical care costs only. Outcomes were estimated over a trial-based time horizon of 24 months and a lifetime time horizon using a monthly cycle. Costs and outcomes were discounted at 3% per year.

© 2019 Whittington MD et al. *JAMA Network Open*.

eFigure. Model Schematic

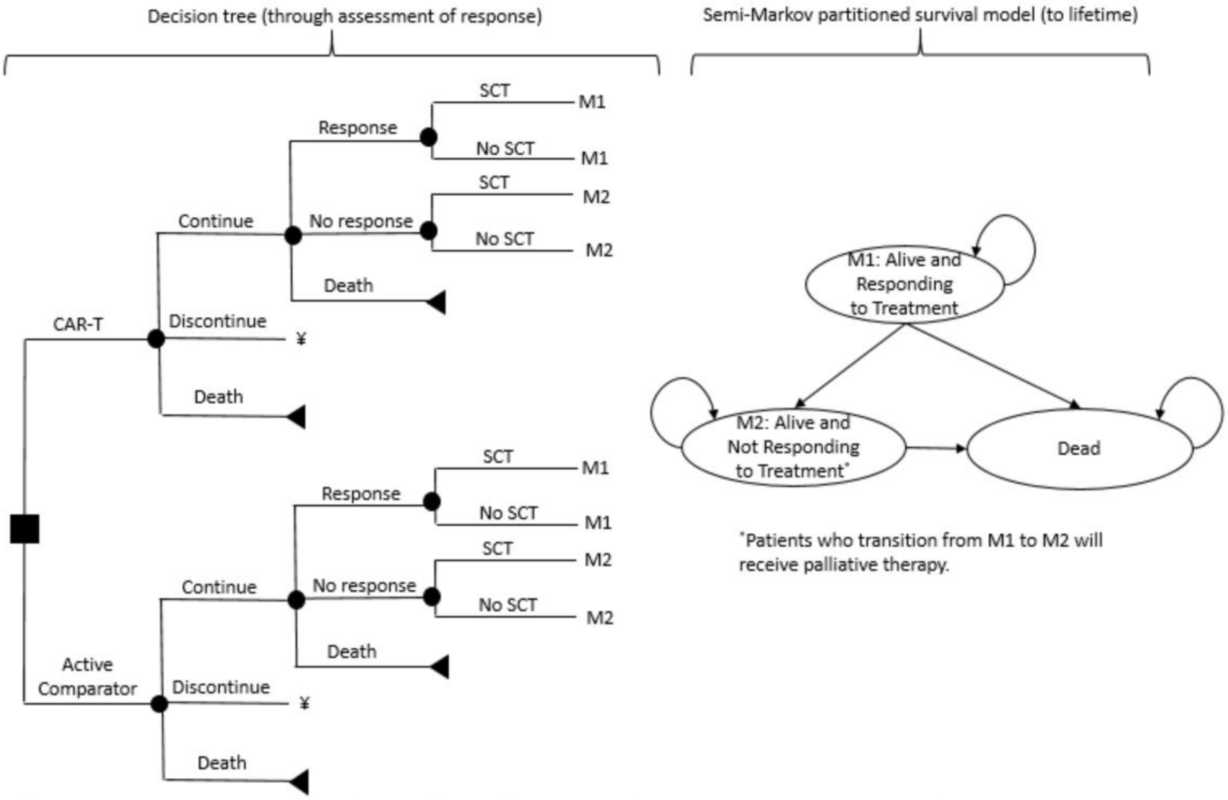

¥Patients who discontinue due to manufacturing failure will transition to the active comparator. Patients who discontinue due to adverse event will receive no further antileukemic/antilymphomic therapy and will enter the semi-Markov partitioned survival model at M2.
CAR-T: Chimeric antigen receptor T-cell therapies
SCT: Stem cell transplantation

© 2019 Whittington MD et al. *JAMA Network Open*.

eTable 1. Key Model Assumptions

Assumption	Rationale
**Stem cell transplantation (SCT), if it occurred, occurred within two months of the model start and no further SCT events were modeled.**	Based on mean time from CAR-T therapy to stem cell transplantation estimated by Lee et al.[3,11]
**Patients received a single full course of CAR-T therapy.**	CAR-T therapies are considered an end-of-line treatment with no clinical evidence on re-treatment.
**All patients who transitioned to the alive and not responding to treatment health state received palliative chemotherapy.**	The intervention and comparator therapies are considered end-of-line treatments.
**Patients who discontinued CAR-T due to an AE before receiving the infusion received no further antilymphomic therapy.**	Those who experienced a severe AE would be unable to tolerate further active therapy.
**Patients who did not receive CAR-T therapy due to a manufacturing failure received the active comparator.**	Those who experienced a manufacturer failure would be able to tolerate further active therapy.
**The model included costs and outcomes associated with grade 3/4 AEs.**	Less severe adverse events are not expected to significantly impact patient health or costs.
**The cost of a hospital admission for treatment administration included the per diem cost for hospital days and the costs of therapies administered during the hospitalization.**	Future bundled payments were assumed to approximate the cost of the resources used under a fee-for-service framework.

© 2019 Whittington MD et al. *JAMA Network Open*.

eTable 2. Treatment Regimens

B-cell Lymphoma	Regimen	Notes	Source
**Axicabtagene Ciloleucel**	2 x 10^6 CAR-T cells/kg		Locke et al., 2017[2]
**Chemotherapy (R-DHAP)**	Dexamethasone 40 mg on days 1-4 + cytarabine 2 g/m^2 every 12h for 2 doses on day 2 + cisplatin 100 mg/m^2 on day 3; every 21 days for three cycles, rituximab 375 mg/m^2 on day 1 of each cycle; an additional rituximab (375 mg/m^2) was given on day -1 of the first cycle		Gisselbrecht et al., 2010[8]
**Bridging chemotherapy**	No bridging chemotherapy used with axicabtagene ciloleucel		Locke et al., 2017[2]
**Lymphocyte depleting chemotherapy**	Fludarabine (30 mg/m^2 IV daily for 3 days) and cyclophosphamide (500 mg/m^2 IV daily for 3 days)	CAR-T treatments only	Locke et al., 2017[2]
**Tocilizumab**	8 mg/kg intravenously over 1 hour (maximum dose 800 mg)	For the management of cytokine release syndrome	Kymriah/Yescarta Package Insert[9]
**Intravenous immunoglobulin**	0.5 g/kg every 4 weeks[3] for 11.4 months[10]	For the management of B-cell aplasia; costs only assigned to those who are alive and responding to treatment	Maude et al., 2017[10]

© 2019 Whittington MD et al. *JAMA Network Open*.

eTable 3. Response to Treatment

B-cell Lymphoma	Axicabtagene Ciloleucel	Chemotherapy
**Percent Achieving Response (Complete or Partial)**	82.0%[2]	26.0%[12]
**Percent Dead Before Assessment of Response**	0.0%[2]	0.0%[12]
**Percent Achieving No Response**	18.0%[2]	74.0%[12]
Note: The denominator is the number of people who received a CAR-T infusion for CAR-T therapies and the number of people who initiated the chemotherapy regimen for comparator therapies.		

© 2019 Whittington MD et al. *JAMA Network Open*.

eTable 4. Source of Kaplan-Meier Curves to Calculate Transition Probabilities

B-cell Lymphoma	Axicabtagene Ciloleucel	Chemotherapy
**Progression-Free Survival**	Progression-free survival curve (Figure 2B) for ZUMA-1[6]	No published progression-free survival curve; therefore, the progression-free survival curve was derived from available overall survival data for SCHOLAR-1 chemotherapies, by assuming the proportional relationship from a published progression-free survival and overall survival curve for R-DHAP in the same disease state.[13]
**Overall Survival**	Overall survival curve (Figure 2C) for ZUMA-1[6]	Figure 3A in SCHOLAR-1[12]

© 2019 Whittington MD et al. *JAMA Network Open*.

eTable 5. Receipt of Stem Cell Transplantation

B-Cell Lymphoma	Axicabtagene Ciloleucel*	Chemotherapy
**Percent That Receive Transplantation**	2.97%; (3/101)[7]	29.9%; (180/603)[12]
*Denominator is the number of patients that received a CAR-T infusion regardless of response status		

© 2019 Whittington MD et al. *JAMA Network Open*.

eTable 6. Included Adverse Event Rates

Grade 3/4 Adverse Event	Axicabtagene Ciloleucel[9]	Chemotherapy[12]
Abdominal Pain	1%	N/R
Acute Kidney Injury	N/R	N/R
B-Cell Aplasia/ Hypogammaglobulinemia*	15%	6.6%[14]
Cytokine Release Syndrome	13%	N/R
Decreased Appetite	2%	N/R
Delirium	6%	N/R
Diarrhea	4%	N/R
Encephalopathy	29%	N/R
Epistaxis	N/R	N/R
Fatigue	3%	9%
Febrile Neutropenia	36%	23%
Headache	1%	N/R
Hypotension	15%	N/R
Hypoxia	11%	N/R
Infections	23%	9%
Nausea	0%	8%
Pain in Extremity	2%	N/R
Petechiae	N/R	N/R
Pyrexia	N/R	N/R
Tachycardia	2%	N/R
Vomiting	1%	7%

N/R: Not reported
*Any grade, not just grades 3 or 4

eTable 7. Model Health State Utilities

B-cell Lymphoma	Utility	Source
**Alive and Not Responding to Treatment**	0.39	Chen et al., 2017[15]
**Alive and Responding to Treatment (i.e. progression-free or event-free survival)**	0.83	Chen et al., 2017[15]
**Long-Term Survivor-Alive, Responding to Treatment after 5 Years (i.e. progression-free or event-free survival)**	0.83	Chen et al., 2017[15]

© 2019 Whittington MD et al. *JAMA Network Open*.

eTable 8. Treatment-Related Disutilities

Health State	Disutility	Notes	Source
**Chemotherapy**	-0.42	Applied for duration of treatment. Applies to pre-CAR-T treatment chemotherapies as well.	Sung et al.[3,18]
**Stem cell transplantation**	-0.57	Applied for duration of decision tree and includes all decrements due to adverse events.	Sung et al.[3,18]

© 2019 Whittington MD et al. *JAMA Network Open*.

eTable 9. Treatment Acquisition Costs for B-Cell Lymphoma Cohort

B-cell Lymphoma	Unit	Price per Unit*
**Axicabtagene Ciloleucel**	2 x 10^6 CAR-T cells/kg	$373,000†
**Dexamethasone**	1mg	$0.33
**Cytarabine**	1mg/1ml	$0.01
**Cisplatin**	1mg/1ml	$0.21
**Rituximab**	1mg/1ml	$8.48
**Fludarabine**	1mg/1ml	$2.10
**Cyclophosphamide**	1mg/1ml	$0.42
**Tocilizumab**	1mg/1ml	$4.37
**Intravenous immunoglobulin**	1mg/1ml	$0.08

*Price as of October 8th, 2017; does not include any potential hospital mark-up

†Represents the total, not unit, wholesale acquisition costs of CAR-T therapy

© 2019 Whittington MD et al. *JAMA Network Open*.

eTable 10. Unit Costs for Health Care Utilization

Cost Parameter	Value	Source
**Cost per Hospital day**	$3,037	HCUP Statistical Brief #125[21]
**Cost per day in ICU**	$5,296	Dasta et al., 2005[22]
**Office Visit**	$74	Physicians' Fee and Coding Guide[23] (HCPCS code 99213)
**Leukapheresis**	$1,093	Physicians' Fee and Coding Guide[23] (HCPCS code 36511)
**Intravenous Treatment Administration (first hour)**	$140	Physicians' Fee and Coding Guide[23] (HCPCS code 96413)
**Intravenous Treatment Administration (each additional hour)**	$29	Physicians' Fee and Coding Guide[23] (HCPCS code 96415)
**Intravenous Treatment Administration (each additional sequence/drug)**	$66	Physicians' Fee and Coding Guide[23] (HCPCS code 96417)
**Hematology Panel**	$11	Physicians' Fee and Coding Guide[23] (HCPCS code 82025)
**Liver Function Test**	$8	Physicians' Fee and Coding Guide[23] (HCPCS code 80076)

© 2019 Whittington MD et al. *JAMA Network Open*.

eTable 11. Administration and Monitoring

Administration and Monitoring for Different Therapies		
**Time Horizon**	**Axicabtagene Ciloleucel**	**Chemotherapy**
**Decision Tree**	Leukapheresis and 15 inpatient hospital days[7]	1 hour of IV administration per cytarabine, cisplatin, and rituximab administration
**Year 1**	12 outpatient visits, 12 complete blood counts, and 6 liver function tests	12 outpatient visits, 12 complete blood counts, and 6 liver function tests
**Years 2-5**	10 outpatient visits, 10 complete blood counts	10 outpatient visits, 10 complete blood counts
**Years 6 to Lifetime**	Average healthcare utilization for age group	Average healthcare utilization for age group

© 2019 Whittington MD et al. *JAMA Network Open*.

eTable 12. Adverse Event Unit Costs

Adverse Event (ICD-9-CM)	Mean ($)	Standard Error ($)
**Abdominal pain (789.0)**	$6,766	$7,148
**Acute kidney injury (584)**	$17,357	$20,817
**Decreased appetite (783.0)**	$9,918	$14,317
**Delirium (780.09)**	$8,284	$11,440
**Diarrhea (787.91)**	$7,880	$10,698
**Encephalopathy (348.30)**	$11,222	$12,165
**Epistaxis (784.7)**	$9,054	$18,629
**Fatigue (780.71)**	$7,486	$11,105
**Febrile neutropenia (288.00)**	$13,975	$22,204
**Headache (784.0)**	$7,130	$7,810
**Hypotension (458.9)**	$8,362	$10,336
**Hypoxia (799.02)**	$8,472	$12,697
**Infections (686.9)**	$7,680	$10,857
**Nausea (787.02)**	$6,229	$7,314
**Pain in extremity (729.5)**	$6,863	$10,172
**Petechiae (782.7)**	$8,303	$12,486
**Pyrexia (780.60)**	$7,401	$9,826
**Tachycardia (785.0)**	$6,885	$9,431
**Vomiting (787.03)**	$5,731	$7,482

© 2019 Whittington MD et al. *JAMA Network Open*.

eReferences

1. Institute for Clinical and Economic Review. CAR-T Therapies: Final Evidence Report and Meeting Summary. 2018; https://icer-review.org/meeting/car-t/. Accessed March 23, 2018.
2. Locke FL, Neelapu SS, Bartlett NL, Lekakis LJ, Miklos D, Go WY. Primary Results from ZUMA-1: A Pivotal Trial of Axicabtagene Ciloleucel (Axi-cel; KTE-C19) in Patients with Refractory Aggressive Non-Hodgkin Lymphoma (NHL). Paper presented at: American Association of Cancer Research Annual Research Meeting 20172017; Washington, DC.
3. Hettle R, Corbett M, Hinde S, et al. The assessment and appraisal of regenerative medicines and cell therapy products: an exploration of methods for review, economic evaluation and appraisal. *Health technology assessment (Winchester, England).* 2017;21(7):1-204.
4. Guyot P, Ades AE, Ouwens MJ, Welton NJ. Enhanced secondary analysis of survival data: reconstructing the data from published Kaplan-Meier survival curves. *BMC medical research methodology.* 2012;12:9.
5. WebPlotDigitizer. http://arohatgi.info/WebPlotDigitizer/app/.
6. Neelapu SS, Locke FL, Bartlett NL, et al. Axicabtagene Ciloleucel CAR T-Cell Therapy in Refractory Large B-Cell Lymphoma. *New England Journal of Medicine.* 2017;377(26):2531-2544.
7. Kite Pharma. Data on file for ZUMA-1.
8. Gisselbrecht C, Glass B, Mounier N, et al. Salvage regimens with autologous transplantation for relapsed large B-cell lymphoma in the rituximab era. *Journal of clinical oncology : official journal of the American Society of Clinical Oncology.* 2010;28(27):4184-4190.
9. YESCARTA (axicabtagene ciloleucel) package insert. 2017.
10. Maude SL, Grupp SA, Pulsipher M. Analysis of Safety Data from 2 Multicenter Trials of CTL019 in Pediatric and Young Adult Patients with Relapsed/Refractory B-Cell Acute Lymphoblastic Leukemia. *Haematologica.* 2017;Abs P517.
11. Lee DW, Kochenderfer JN, Stetler-Stevenson M, et al. T cells expressing CD19 chimeric antigen receptors for acute lymphoblastic leukaemia in children and young adults: a phase 1 dose-escalation trial. *Lancet (London, England).* 2015;385(9967):517-528.
12. Crump M, Neelapu SS, Farooq U, et al. Outcomes in refractory diffuse large B-cell lymphoma: results from the international SCHOLAR-1 study. *Blood.* 2017.
13. Schirmbeck NG, Mey UJ, Olivieri A, et al. Salvage Chemotherapy with R-DHAP in Patients with Relapsed or Refractory Non-Hodgkin Lymphoma. *Cancer investigation.* 2016;34(8):361-372.
14. Casulo C, Maragulia J, Zelenetz AD. Incidence of hypogammaglobulinemia in patients receiving rituximab and the use of intravenous immunoglobulin for recurrent infections. *Clinical lymphoma, myeloma & leukemia.* 2013;13(2):106-111.
15. Chen Q, Staton AD, Ayer T, Goldstein DA, Koff JL, Flowers CR. Exploring the potential cost-effectiveness of precision medicine treatment strategies for diffuse large B-cell lymphoma. *Leukemia & lymphoma.* 2017:1-10.
16. Doorduijn J, Buijt I, van der Holt B, Steijaert M, Uyl-de Groot C, Sonneveld P. Self-reported quality of life in elderly patients with aggressive non-Hodgkin's lymphoma

© 2019 Whittington MD et al. *JAMA Network Open*.

treated with CHOP chemotherapy. *European Journal of Haematology*. 2005;75(2):116-123.
17. Sullivan PW, Ghushchyan V. Preference-Based EQ-5D Index Scores for Chronic Conditions in the United States. *Medical decision making : an international journal of the Society for Medical Decision Making.* 2006;26(4):410-420.
18. Sung L, Buckstein R, Doyle JJ, Crump M, Detsky AS. Treatment options for patients with acute myeloid leukemia with a matched sibling donor: a decision analysis. *Cancer.* 2003;97(3):592-600.
19. Pelletier EM, Smith PJ, Dembek CJ. Payer Costs of Autologous Stem Cell Transplant: Results from a U.S. Claims Data Analysis. *Blood.* 2008;112(11):2373-2373.
20. Maziarz RT, Hao Y, Guerin A, et al. Economic burden following allogeneic hematopoietic stem cell transplant in patients with diffuse large B-cell lymphoma. *Leukemia & lymphoma.* 2017:1-10.
21. Price RA, Stranges E, Elixhauser E. Cancer Hospitalizations for Adults, 2009. *HCUP Statistical Brief #125.* 2012.
22. Dasta JF, McLaughlin TP, Mody SH, Piech CT. Daily cost of an intensive care unit day: the contribution of mechanical ventilation. *Critical care medicine.* 2005;33(6):1266-1271.
23. Centers for Medicare and Medicaid Services. Physician Fee Schedule Search. 2017; https://www.cms.gov/apps/physician-fee-schedule/license-agreement.aspx.
24. Department of Health and Human Services: Agency for Healthcare Research and Quality. HCUPnet: Healthcare Cost and Utilization Project. https://hcupnet.ahrq.gov/.

© 2019 Whittington MD et al. *JAMA Network Open*.
